# FilGAP, a Rac-specific Rho GTPase-activating protein, is a novel prognostic factor for follicular lymphoma

**DOI:** 10.1002/cam4.423

**Published:** 2015-01-29

**Authors:** Tatsuya Nishi, Hiroyuki Takahashi, Miki Hashimura, Tsutomu Yoshida, Yasutaka Ohta, Makoto Saegusa

**Affiliations:** 1 Department of Pathology, Kitasato University School of Medicine1-15-1 Kitasato, Minami-ku, Sagamihara, Kanagawa, 252-0374, Japan; 2 Division of Cell Biology, Department of Biosciences, Kitasato University School of Science1-15-1 Kitasato, Minami-ku, Sagamihara, Kanagawa, 252-0374, Japan

**Keywords:** B-lymphocyte, FilGAP, follicular lymphoma, prognosis, Rac

## Abstract

FilGAP, a Rho GTPase-activating protein (GAP), acts as a mediator of Rho/ROCK (Rho-associated protein kinase)-dependent amoeboid movement, and its knockdown results in Rac-driven mesenchymal morphology. Herein, we focus on the possible roles of FilGAP expression in normal and malignant lymphocytes. Eighty-three cases of follicular lymphoma (FL), 84 of diffuse large B-cell lymphoma (DLBCL), and 25 of peripheral T-cell lymphoma (PTCL), as well as 10 of normal lymph nodes, were immunohistochemically investigated. In normal lymph nodes, FilGAP immunoreactivity was significantly higher in lymphocytes in the mantle zone as compared to those in the germinal center and paracortical areas. In contrast, the expression levels of both cytoplasmic and perinuclear Rac1 were significantly lower in the germinal center as compared to paracortical regions, suggesting that changes in the FilGAP/Rac axis may occur in B-cell lineages. In malignant lymphomas, FilGAP expression was significantly higher in B-cell lymphomas than PTCL, and the immunohistochemical scores were positively correlated with cytoplasmic Rac1 scores in FL and DLBCL, but not in PTCL. Patients with FL and germinal center B-cell-like (GCB)-type DLBCL showing high FilGAP scores had poor overall survival rates as compared to the low-score patients. Moreover, multivariate Cox regression analysis showed that a high FilGAP score was a significant and independent unfavorable prognostic factor in FL, but not in DLBCL. In conclusion, FilGAP may contribute to change in cell motility of B-lymphocytes. In addition, its expression appears to be useful for predicting the behavior of B-cell lymphoma, in particular FL.

## Introduction

Follicular lymphoma (FL) is the most common type of indolent B-cell lymphoma, accounting for 10–20% of all malignant lymphomas (MLs) 1. FL is characterized by frequent relapses and progression to treatment-resistant disease or transformation to high-grade feature, despite initial responsiveness to chemotherapy or radiotherapy 2,3. For risk assessment, the Follicular Lymphoma International Prognostic Index (FLIPI) has been developed on the basis of five variables, including age, Ann Arbor stage, hemoglobin levels, number of nodal site areas, and serum low-density lipoprotein (LDH) 4, in contrast to the International Prognostic Index (IPI) for aggressive MLs 5. The low-, intermediate-, and high-risk groups are identified with 10-year overall survival (OS) rates of 71%, 51%, and 36%, respectively 6. Thus, clinical data represent the major source of prognostic information, but many biological factors are not yet clinically validated or easily assessable.

Abnormal cell migration and invasion are characteristics of malignant cells, being closely associated with tumor progression. Members of the Rho GTPase family, including RhoA, Rac, and Cdc42, are key regulators of cell migration by modulating mesenchymal and amoeboid motility 7,8. Amoeboid movement is most commonly observed in MLs and small cell lung carcinomas, allowing tumor cells to undergo early detachment and metastatic spread from primary tumors. In contrast, cells with mesenchymal migration, such as fibrosarcomas and glioblastomas, have a fibroblast-like spindle-shaped morphology that is dependent on integrin-mediated adhesion dynamics 9.

FilGAP is a Rho GTPase-activating protein (GAP) and binds to the actin filament cross-linking protein filamin A (FLNa) 10–12. Knockdown of endogenous FilGAP induces a Rac-driven elongated mesenchymal morphology, while its overexpression results in membrane blebbing and a rounded amoeboid morphology, indicating that FilGAP mediates antagonism between Rac and Rho that suppresses cell protrusion and promotes cell contraction 13,14. However, little is known about possible roles of FilGAP in MLs.

In this study, we investigated the expression of FilGAP, with reference to the status of its associated molecules, including FLNa, integrin *β*2, epithelial cell transforming factor 2 (ECT2), and Rac1, in FL, diffuse large B-cell lymphoma (DLBCL), and peripheral T-cell lymphoma (PTCL). In addition, we also examined whether FilGAP is suitable as an independent prognostic factor of MLs.

## Materials and Methods

### Clinical cases

A total of 192 cases of MLs, including 83 of FL, 84 of DLBCL, and 25 of PTCL newly diagnosed between 1998 and 2012 in Kitasato University hospital, were selected for this retrospective study. All tumor tissues were obtained from each patient by lymph node biopsy. Pathological diagnosis was made according to the criteria of the World Health Organization Classification (2008) 1. All patients were treated with a combination of chemotherapy such as CHOP or CHOP-like regimes, with or without rituximab. All clinical and laboratory data, along with the follow-up data, were obtained from the hospital's medical records and patient charts and then FLIPI for FL and IPL for DLBCL were evaluated (Table S1). Ten biopsy specimens of non-neoplastic lymph nodes were also investigated. All tissues were routinely fixed in 10% formalin and processed for embedding in paraffin wax. In addition, a variety of organ tissues obtained from five autopsy cases were snap-frozen in liquid nitrogen for reverse transcription polymerase chain reaction (RT-PCR) and western blot assays. Approval for this study was given by the Ethics Committee of the Kitasato University School of Medicine (B13-29).

### Immunohistochemistry

Immunohistochemistry (IHC) was performed using a combination of microwave oven heating and polymer immunocomplex (Envision, Dako, Glostrup, Denmark) methods, with the following primary antibodies: CD3, CD5, CD10, CD20, CD79a, Bcl2, Bcl6, and MUM1, all from Dako. Both FLNa and integrin *β*2 were from Millipore (Billerica, MA). ECT2 and Rac 1 were from Santa Cruz Biotechnology (Santa Cruz, CA) and BD Bioscience (San Jose, CA), respectively. Rabbit polyclonal anti-FilGAP antibody was developed as described previously 10. To evaluate the specificity of the FilGAP antibody in IHC analysis, absorption assay was performed, as described previously 15. Briefly, the primary antibody was incubated for 1 h with excess purified FilGAP antigen, and then IHC analysis was carried out.

For evaluation of the IHC findings, cases were considered as positive when more than 30% of tumor cells were stained, on the basis of the methods reported by Hans et al. 16. With respect to the immunophenotype of DLBCL, cases positive for CD10 and Bcl6 and negative for MUM1 were regarded as having a germinal center-like (GCB) phenotype and others as non-GCB type (Table S1), as described by Hans et al. 16. Immunoreactivity scores for FilGAP, FLNa, integrin *β*2, ECT2, and cytoplasmic Rac 1 were also calculated by multiplying the percentage of immunopositive cells by the immunointensity values, as described previously 17. Perinuclear Rac1 grade was subdivided into four categories as follows: 0, negative; 1, weak immunointensity; 2, moderate; and 3, strong. The perinuclear immunointensity of lymphocytes at paracortical regions, used as internal controls, was designated as strong. In addition, perinuclear Rac1 score was calculated by multiplying the percentage of perinuclear immunopositive cells by the perinuclear grade.

### In situ hybridization

Riboprobes for FilGAP containing nucleotides 1027 to 1726 of the *FilGAP* gene were generated by in vitro transcription, using full length FilGAP cDNA 10, and in situ hybridization (ISH) assays were performed using the GenPoint Tyramide Signal Amplification System (Dako), as described previously 18. Cases with more than 10% cells positive for ISH signals were defined as positive.

### RT-PCR

cDNA was synthesized from 2 *μ*g of total RNA and amplification was carried out using specific forward primers for the *FilGAP* gene as follows: variant *(v)1 primer located in exon 3*: 5'-ATCCCTGCAATGAAGAGAACCC-3', *v2 in exon 6*: 5'-TACGATGCCTGAAGACCGGAAT-3', and *v3 in exon 4*: 5'-TGCGTAGACCAGACCAGTGAC-3'. Common reverse primer located in exon 7 was 5'-GAGCCAGACGGTTCCCATATC-3' (Fig. 2A). Primers for the *GAPDH* gene were also applied as internal control, as described previously 17.

### Western blot assays

Total cellular proteins were harvested using 2× Laemmli sample buffer. Aliquots of the proteins were resolved by SDS-PAGE (sodium dodecyl sulfate polyacrylamide gel electrophoresis), transferred to membranes, and probed with primary antibodies, coupled with the ECL detection system (Amersham Pharmacia Biotechnology, Tokyo, Japan).

### Statistics

Comparative data were analyzed using the Mann–Whitney *U* test and the Pearson's correlation coefficient. OS was calculated as the time between onset and death or the date of the last follow-up evaluation. Progression-free survival (PFS) was also examined from the onset of treatment until relapse, disease progression, or last follow-up evaluation. OS and PFS were estimated using the Kaplan–Meier methods, and the statistical comparisons were made using the log-rank test. Univariate and multivariate analyses were performed using the Cox proportional hazards regression model. The cut-off for statistical significance was set as *P* < 0.05.

## Results

### Immunohistochemical specificity of the anti-FilGAP antibody

**Figure 1:**
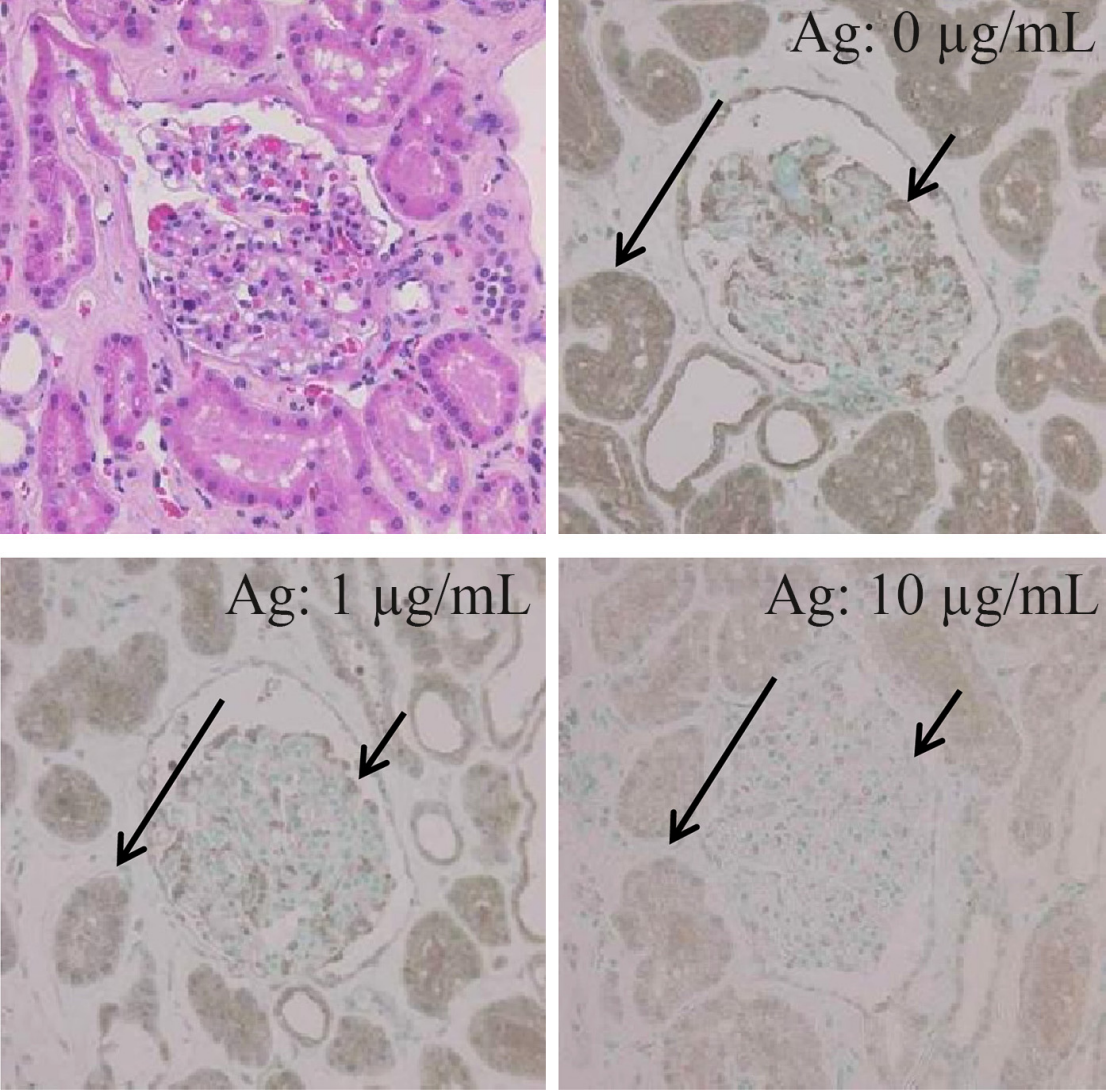
Absorption assay for anti-FilGAP antibody. By pretreatment of the anti-FilGAP antibody with various amounts (0, 1, and 10 *μ*g/mL) of GST (Glutathione S-transferase) fusion protein containing residues 552–748 of FilGAP as antigen (Ag); the immunoreactivity in podocytes (indicated by short arrows) and tubules (indicated by long arrows) is dramatically decreased and/or disappeared. Original magnification, 200×. FilGAP, a Rho GTPase-activating protein (GAP).

To examine the specificity of the anti-FilGAP antibody on formalin-fixed and paraffin-embedded sections, absorption assay was performed using normal human kidney tissues which have the highest expression in podocytes 10,19. Conventional IHC assay revealed distinct cytoplasmic FilGAP immunoreaction in both podocytes and tubules, but such immunoreactivity was decreased and/or disappeared by pretreatment with excess FilGAP antigen (Fig. 1).

### FilGAP expression in normal human tissues

**Figure 2:**
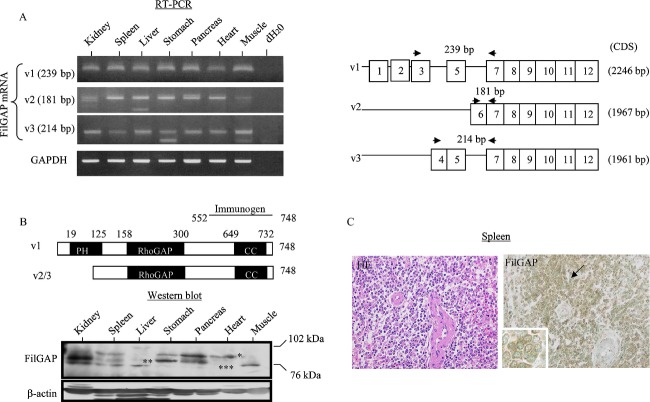
FilGAP expression in normal human tissues. (A) *Left*: Analysis of endogenous FilGAP mRNA expression by RT-PCR assay in a variety of human tissues. *Right*: Schematic representation of primer design for each FilGAP mRNA variant (v). CDS, coding sequence. (B) *Upper*: A schematic diagram of FilGAP v1 and v2/3 isoforms. PH, pleckstrin homology; CC, coiled-coil domain. *Lower*: Analysis of endogenous FilGAP protein expression by western blot assay in a variety of human tissues. Upper (indicated by one asterisk) and lower bands (indicated by two and three asterisks) demonstrate the v1 and v2/3 isoforms. (C) Staining by hematoxylin and eosin (HE) and IHC for FilGAP in the spleen. Note the strong immunopositivity in lymphocytes (indicated by arrows and magnified in the insets) in the white pulp. Original magnification, 200× and 400× (inset). FilGAP, a Rho GTPase-activating protein (GAP); RT-PCR, reverse transcription polymerase chain reaction; IHC, immunohistochemistry.

Several FilGAP isoforms produced by alternative splicing are ubiquitously expressed in most cells and tissues 10,20,21. To examine the expression patterns of FilGAP isoforms in a variety of human tissues, RT-PCR and western blot assays were carried out. As shown in Figure 2A, full length (v1) and shorter mRNA variants lacking the N-terminal pleckstrin homology (PH) domain (v2 and v3) were detected in all tissues investigated. In contrast, the western blot results demonstrated the presence of various protein isoforms at varying expression levels that diverge from the RT-PCR findings (Fig. 2B). By IHC analyses, higher levels of FilGAP immunoreactivity were observed in lymphocytes in the spleen (Fig. 2C), as well as podocytes and tubules in the kidney (Fig. 1).

### Expression of FilGAP and its associated molecules in normal and malignant lymphocytes

**Figure 3:**
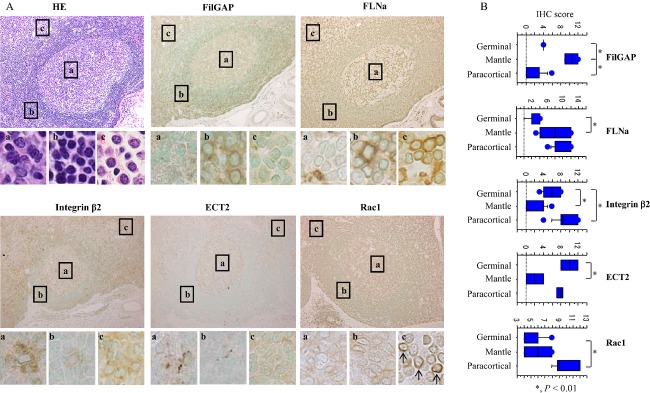
Expression of FilGAP and its associated molecules in normal lymph nodes. (A) Staining by hematoxylin and eosin (HE) and IHC for FilGAP, FLNa, integrin *β*2, ECT2, and Rac1 in the follicular region of lymph nodes. Boxes enclose a magnified view of the (a) germinal center, (b) mantle, and (c) paracortical regions. Note the perinuclear Rac1 immunoreactivity in the paracortical region (indicated by arrows). Original magnification, 200× and 400× (inset). (B) IHC scores for FilGAP, FLNa, integrin *β*2, ECT2, and cytoplasmic (cyto) Rac1 in the germinal center, mantle, and paracortical regions. The data shown are mean ± SD. FilGAP, a Rho GTPase-activating protein (GAP); IHC, immunohistochemistry; FLNa, filamin A; ECT2, epithelial cell transforming factor 2.

On the basis of the above findings, we further examined FilGAP expression in normal and malignant lymphocytes. Representative IHC findings for FilGAP, FLNa, integrin *β*2, ECT2, and Rac1 in normal lymph nodes are illustrated in Figure 3A. Immunopositivity for these molecules was mainly observed in cytoplasmic compartments of lymphocytes. Perinuclear Rac1 immunoreactivity was also observed in lymphocytes with relatively intense cytoplasmic staining, and was found to have significant positive correlation with cytoplasmic Rac1 status (Fig. S1A and Table S2). FilGAP scores were significantly higher in the mantle zone as compared to those in the germinal center and paracortical regions. FLNa scores showed stepwise increases from the germinal center, through the mantle, to the paracortical region. ECT2 scores were significantly higher in the germinal center as compared to those in the mantle zone, in contrast to the significantly higher integrin *β*2 and cytoplasmic and perinuclear Rac1 scores in the paracortical region (Figs.3B and S1A).

Overall, average FilGAP scores were negatively correlated with those for integrin *β*2, ECT2, and cytoplasmic Rac1, as well as perinuclear Rac1 grades (*r* = −0.38, *P* = 0.01). Cytoplasmic Rac1 scores were also positively related to FLNa and integrin *β*2 scores. By evaluating the immunostaining according to the two regions, follicles and paracortex, FilGAP scores showed strong positive correlation with FLNa scores in both categories, in contrast to a lack of association with cytoplasmic Rac1 scores (Table1).

**Table 1:** Correlations among several FilGAP-related markers investigated in normal lymph node

	FilGAP *r* (*P*)	FLNa *r* (*P*)	Integrin *β*2 *r* (*P*)	ECT2 *r* (*P*)
Overall (follicular and paracortical areas)				
Filamin A	0.13 (0.82)	NE	NE	NE
Integrin *β*2	−0.62 (<0.01)	0.23 (0.17)	NE	NE
ECT2	−0.64 (0.05)	−0.24 (0.48)	0.6 (0.07)	NE
Rac1	−0.37 (0.09)	0.49 (0.03)	0.68 (<0.01)	0.08 (0.8)
Follicular area (B-lymphocytes)				
Filamin A	0.71 (<0.01)	NE	NE	NE
Integrin *β*2	−0.49 (0.06)	−0.37 (0.11)	NE	NE
ECT2	−0.79 (0.03)	−0.59 (0.17)	0.8 (0.06)	NE
Rac1	0.09 (0.5)	−0.81 (0.79)	0.29 (0.23)	−0.07 (0.74)
Paracortical area (T-lymphocytes)				
Filamin A	0.85 (<0.01)	NE	NE	NE
Integrin *β*2	0.11 (0.87)	0.33 (0.25)	NE	NE
ECT2	0.61 (0.38)	0.55 (0.34)	−0.84 (0.38)	NE
Rac1	0.45 (0.31)	0.41 (0.29)	0.7 (0.17)	−1 (0.31)

FilGAP, a Rho GTPase-activating protein (GAP); FLNa, filamin A; NE, not examined; ECT2, epithelial cell transforming factor 2; *r*, Pearson's correlation coefficient.

**Figure 4:**
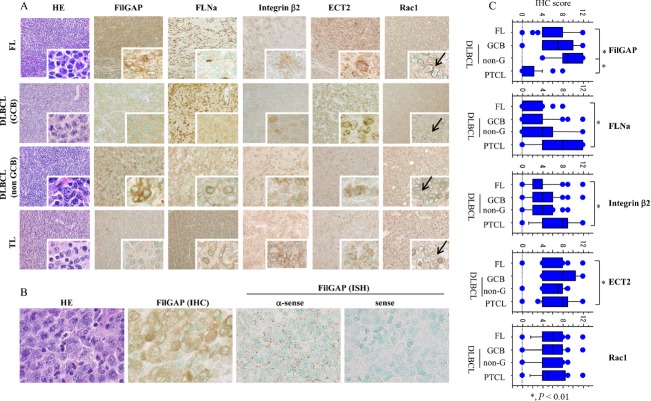
Expression of FilGAP and its associated molecules in malignant lymphomas. (A) Staining by hematoxylin and eosin (HE) and IHC for FilGAP, FLNa, integrin *β*2, ECT2, and Rac1 in FL, GCB, and non-GCB-type DLBCLs, and PTCL. Cytoplasmic immunoreactivity for these markers in lymphoma cells is magnified in the insets. Note the perinuclear Rac1 immunoreactivity in the lymphoma cells (indicated by arrows). Original magnification, 200× and 400× (inset). (B) Staining by HE and IHC for FilGAP protein, and ISH for its mRNA in DLBCL. Note the positive FilGAP mRNA signals in the lymphoma cells, consistent with the strong immunoreactivty. Original magnification, 400×. (C) IHC scores for FilGAP, FLNa, integrin *β*2, ECT2, and cytoplasmic (Cyto) Rac1 in FL, GCB-type DLBCL, non-GCB-type DLBCL, and PTCL. The data shown are mean ± SD. FilGAP, a Rho GTPase-activating protein (GAP); IHC, immunohistochemistry; FLNa, filamin A; FL, follicular lymphoma; GCB, germinal center B-cell-like; DLBCL, diffuse large B-cell lymphoma; PTCL, peripheral T-cell lymphoma; ISH, in situ hybridization; ECT2, epithelial cell transforming factor 2.

Representative IHC findings for FilGAP, FLNa, integrin *β*2, ECT2, and Rac1 in MLs are illustrated in Figure 4A. The immunoreaction patterns of these markers were similar to those in normal lymphocytes. Perinuclear Rac1 immunoreactivity was also observed frequently in all ML categories, and cytoplasmic Rac1 scores were found to be significantly correlated with both perinuclear Rac1 grades and scores (Fig. S1B and Table S2). In 10 cases of FL and 9 of DLBCL, positivity for FilGAP mRNA signals as detected by ISH assay was significantly associated with the immunoreactivity in most cases (Fig. 4B), indicating that its expression may be mainly regulated at the transcriptional level.

Average FilGAP scores were significantly higher in B-cell lymphomas than those in PTCL, while FLNa, integrin *β*2, and ECT2 scores were significantly higher in PTCL than those in FL. There were no significant differences in cytoplasmic Rac1 scores among any categories (Fig. 4C). As shown in Table2, FilGAP scores were positively correlated with cytoplasmic Rac1 scores in both FL and DLBCL, along with significantly positive correlation with perinuclear Rac1 grade (*r *= 0.47, *P* = 0.02) in the former. Cytoplasmic Rac1 scores were also positively correlated with FLNa scores in FL and GCB-type DLBCL, and negatively to ECT2 scores in PTCL. In addition, the FilGAP score was significantly higher (*P* < 0.01) in FL than that in normal B-lymphocytes located at the germinal center, while such findings were not observed for the cytoplasmic Rac1 score (data not shown).

**Table 2:** Correlations among several FilGAP-related markers investigated in malignant lymphoma

	FilGAP *r* (*P*)	FLNa *r* (*P*)	Integrin *β*2 *r* (*P*)	ECT2 *r* (*P*)
FL				
Filamin A	0.27 (0.03)	NE	NE	NE
Integrin *β*2	0.03 (0.95)	0.24 (0.07)	NE	NE
ECT2	0.04 (0.78)	−0.2 (0.12)	0.12 (0.38)	NE
Rac1	0.56 (<0.01)	0.57 (<0.01)	−0.12 (0.61)	−0.05 (0.58)
DLBCL (GCB)				
Filamin A	0.24 (0.24)	NE	NE	NE
Integrin *β*2	−0.12 (0.57)	−0.13 (0.43)	NE	NE
ECT2	0.4 (0.02)	0.19 (0.27)	0.13 (0.45)	NE
Rac1	0.79 (0.03)	0.67 (0.08)	−0.24 (0.31)	0.6 (0.2)
DLBCL (non-GCB)				
Filamin A	−0.08 (0.27)	NE	NE	NE
Integrin *β*2	−0.2 (0.34)	0.03 (0.86)	NE	NE
ECT2	0.11 (0.25)	−0.09 (0.59)	0.18 (0.25)	NE
Rac1	0.58 (0.04)	−0.07 (0.98)	−0.17 (0.67)	0.39 (0.31)
PTCL				
Filamin A	0.27 (0.22)	NE	NE	NE
Integrin *β*2	−0.11 (0.67)	0.14 (0.59)	NE	NE
ECT2	0.27 (0.13)	−0.21 (0.35)	0.37 (0.18)	NE
Rac1	−0.13 (0.26)	0.14 (0.83)	−0.19 (0.69)	−0.66 (<0.01)

FilGAP, a Rho GTPase-activating protein (GAP); FLNa, filamin A; *r*, Pearson's correlation coefficient; FL, follicular lymphoma; DLBCL, diffuse large B-cell lymphoma; ECT2, epithelial cell transforming factor 2; GCB, germinal center B-cell-like; PTCL, peripheral T-cell lymphoma; NE, not examined.

### Associations of FilGAP expression with clinicopathological factors and prognosis in MLs

To evaluate the clinicopathological and prognostic significance of FilGAP expression, FilGAP scores were divided into two categories (high and low) on the basis of the mean + SD values (9, 9, 11, and 3 in FL, GCB-type DLBCL, non-GCB-type DLBCL, and TCL, respectively) as the cut-off.

There were no significant differences in age, sex, clinical stage, FLIPI or IPI category, or nuclear grade between low and high FilGAP score groups in any categories (Table S3). No differences in the regimens and courses of chemotherapy were also evident between the two groups (Table S3).

**Figure 5:**
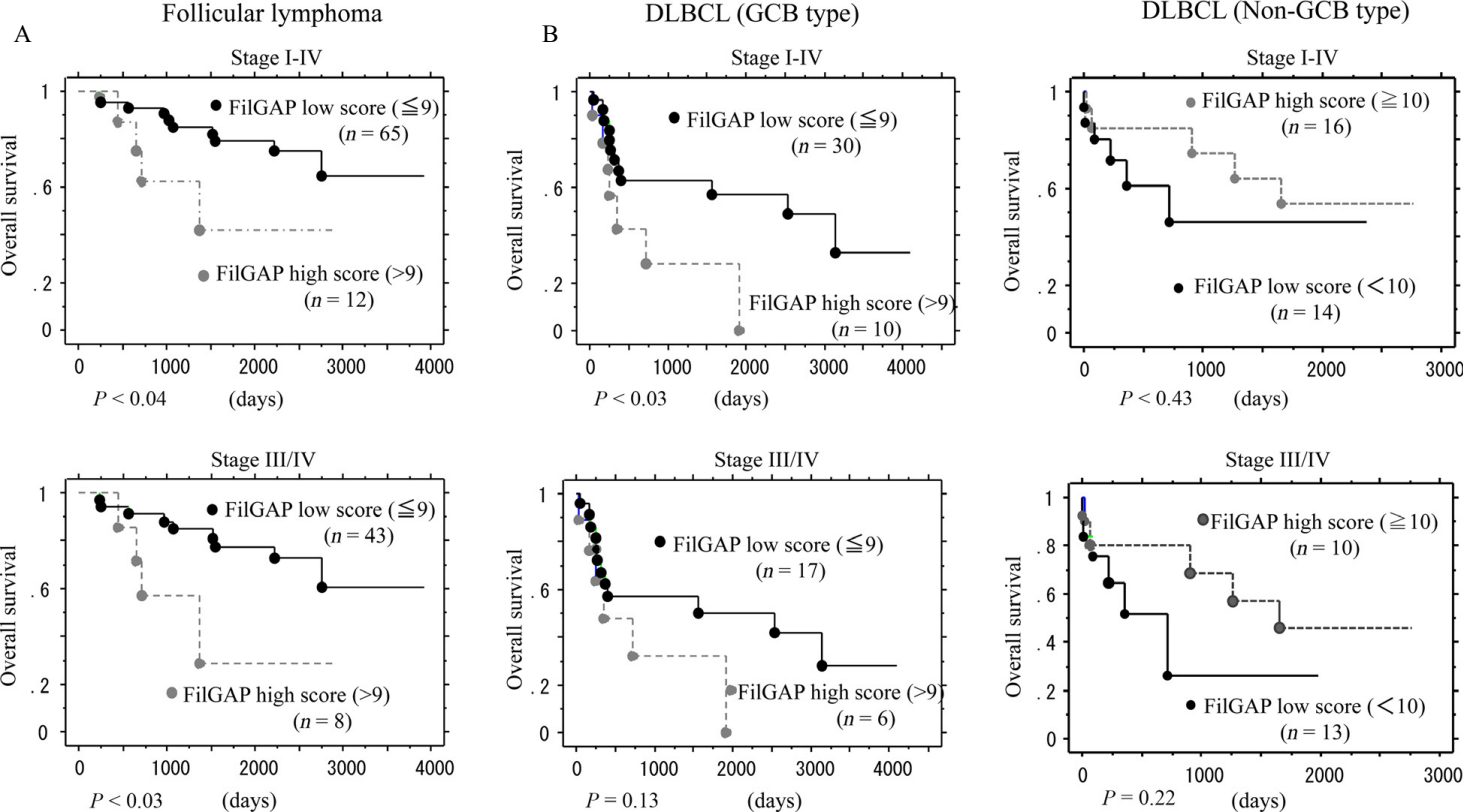
Overall survival (OS) and FilGAP expression. (A) OS of stage I–IV (upper) and III/IV (lower) FL patients based on FilGAP expression. (B) OS of stage I–IV (upper) and III/IV (lower) in GCB- (left) and non-GCB- (right) type DLBCL patients based on FilGAP expression. FilGAP, a Rho GTPase-activating protein (GAP); FL, follicular lymphoma; GCB, germinal center B-cell-like; DLBCL, diffuse large B-cell lymphoma.

Kaplan–Meier curves for survival rates with respect to the FilGAP expression status are shown in Figure 5. Both stage I–IV and III/IV patients with FL who displayed high FilGAP scores had poorer OS rates as compared to the low FilGAP score patients. Similar associations were also observed in GCB, but not non-GCB type, DLBCL, although the difference did not reach significance in patients with stage III/IV disease, probably due to the relatively small number of cases investigated. There were no significant differences in both 2 and 5 year PFS rates between low- and high-FilGAP score patients with FL or DLBCL (Fig. S2). No significant differences in OS rates between low and high scores of integrin *β*2, ECT2, and cytoplasmic Rac1 were evident in stage I–IV patients of FL when cases were subdivided on the basis of the mean + SD values (Fig. S3).

Univariate Cox proportional hazards regression analysis was used to assess the following prognostic factors. As shown in Table3, FilGAP score, FLIPI category, and histopathological grade were significant prognostic factors for stage I–IV and/or III/IV FL, while only FilGAP score was significantly associated with prognosis in stage I–IV GCB-type DLBCL. Multivariate Cox regression analysis showed that FilGAP score was a significant and independent prognostic factor for stage I–IV, but not III/IV, FL (Table4). Such associations were not observed for PTCL (data not shown).

**Table 3:** Univariate analyses for several prognostic factors in follicular lymphoma and DLBCL

	Follicular lymphoma				DLBCL (GCB type)			
	Stage I–IV		Stage III/IV		Stage I–IV		Stage III/IV	
	*n*	*P*-value	*n*	*P*-value	*n*	*P*-value	*n*	*P*-value
Gender								
Male	32	0.23	26	0.75	21	0.75	15	0.01
Female	45		25		19		8	
Age								
<60	42	0.21	30	0.21	15	0.06	9	0.48
≧60	35		21		25		14	
FLIPI/IPI ratio								
High/High-in	15	0.05	15	0.05	19	0.7	18	0.71
Low/Low-in	22		22		7		4	
Grade								
Grade 1/2	48	0.39	35	0.03	NE	NE	NE	NE
Grade 3	25		13		NE		NE	
FilGAP								
High	12	0.04	8	0.03	10	0.01	6	0.18
Low	65		43		30		17	

DLBCL, diffuse large B-cell lymphoma; GCB, germinal center B-cell-like; FLIPI, Follicular Lymphoma International Prognostic Index; IPI, International Prognostic Index; Low-in, low intermediate; High-in, high intermediate; FilGAP, a Rho GTPase-activating protein (GAP); NE, not examined; n, number of cases.

**Table 4:** Multivariate analyses for several prognostic factors in follicular lymphoma

	Stage I–IV			Stage III/IV		
	Hazard ratio	95% CI	*P*-value	Hazard ratio	95% CI	*P*-value
Grade	–	–	–	0.37	0.06–2.32	0.29
FLIPI	0.12	0.02–1.06	0.06	0.15	0.01–1.37	0.09
FilGAP	4.90	1.03–23.12	0.04	5.00	0.67–37.67	0.11

CI, confidence interval; FLIPI, Follicular Lymphoma International Prognostic Index; FilGAP, a Rho GTPase-activating protein (GAP).

## Discussion

This study clearly provided evidence that cytoplasmic Rac1 immunoreactivity was frequently observed in normal and malignant lymphocytes. In addition, the perinuclear staining appeared to be relatively common in such cells, with significant association between perinuclear and cytoplasmic Rac1 status. Interestingly, activated Rac1 has been reported to relocalize to the plasma membrane and perinuclear vesicles 22–24. Given that, Rac1 protein levels are posttranscriptionally regulated either by an increase in RNA stability, translation efficiency, and /or protein stability in glioblastomas 24, it appears that subcellular localization of Rac1 may also be an important factor for determination of its functional status.

Consistent with the present data, a rapidly growing body of evidence indicates that FilGAP is expressed ubiquitously in most cells and tissues, with the highest expression in kidney, particularly in podocytes 10,19. Our findings also demonstrated significantly higher FilGAP expression in lymphocytes in the mantle zone as compared to those in the germinal center and paracortical areas, and were positively correlated with that of FLNa, as well as perinuclear Rac1 status, but negatively to that of ECT2. The interaction of FilGAP with FLNa emerges as a key factor in maintaining low levels of active Rac in mechanically challenged cells 25, while ECT2 is capable of mediating GTP exchange on Rac1 during mesenchymal migration and invasion 26. Given the evidence for a close association between decreased FilGAP expression and induction of amoeboid–mesenchymal transition (AMT) 13,27, it is possible that AMT-like features may occur in B-cell lineages through alteration in the Rac1/FilGAP axis in the lymph nodes, particularly in the follicular region. In fact, elongated and enlarged lymphocytes are frequently observed in the germinal center area, in contrast to the presence of small round cells in the mantle zone.

Integrins form an essential mechanical linkage between extracellular and intracellular environments, with *β*-integrin tails connecting to the actin cytoskeleton by binding directly to FLNa 9. Mechanical strain increases *β*-integrin binding to FLNa, whereas it causes FilGAP to dissociate from FLNa 28. In general, amoeboid tumor cells have a low degree of adhesiveness for collagens, due to their low integrin expression. In addition, lymphocytes and leukocytes are considered to be completely or partially independent on the integrin-mediated adhesion system for cell migration 9. Our results showed that the FilGAP score was negatively correlated with integrin *β*2 status in normal lymph nodes, with the exception of the paracortical region. In addition, a lack of such association was also evident in all of the ML categories, indicating that integrin *β*2-related signaling may have a relatively minor role in the FilGAP-FLNa-actin network in normal and malignant lymphoid tissues.

An interesting finding in this study was that three alternative splicing variants of the FilGAP mRNA were detected in all human tissues investigated, in contrast to the variable expression levels of the protein isoforms, which suggests posttranslational modification of the FilGAP protein. In fact, it was demonstrated that the short FilGAP splice isoform (p73RhoGAP2/RC-GAP72) was expressed in only vascular smooth muscle and endothelial cells, while its mRNA was frequently detected in a variety of tissues 21,29. In addition, the shorter isoforms lacking functional PH domain could influence cell motility at the level of phosphoinositide metabolism, since the domains are strongly similar to those of Akt, cytohesin, and GRP1 (general receptor for phosphoinositides-1) 30. It was recently shown that FilGAP is recruited to the plasma membrane by binding to activated small GTP-binding protein Arf6 through the PH domain 31.

An unexpected finding in this study was that FilGAP and /or FLNa scores were positively correlated with cytoplasmic Rac1 scores in B-cell lymphomas, which is inconsistent with idea that the FilGAP/FLNa system suppresses Rac1 activity 9–11. Although we are presently unable to provide an appropriate explanation for this observation, it appears that the regulatory mechanism for Rac1/FilGAP/FLNa axis may be very complex in B-cell lymphomas. Since our findings demonstrated significantly higher FilGAP expression in FL as compared to that in the B-lymphocytes located at the germinal center region, in contrast to no changes in Rac1 expression, it is possible that increased expression of FilGAP relative to Rac1 may be due to activation of FilGAP/FLNa system in FL. This complexity is also supported by the evidence that Rac1-GTP binds to the PH domain of Dbs, a RhoA GEF (guanine nucleotide exchange factor), and stimulates its catalytic activity, leading to RhoA activation in certain cell types 32–34.

To the best of our knowledge, this is the first immunohistochemical analysis of FilGAP expression in MLs to delineate its relationship with prognosis. Although there were no associations between several clinicopathological factors and FilGAP expression, the OS of stage I–IV and/or III/IV patients with FL or GCB-type DLBCL showing high FilGAP scores were significantly poorer than that of FilGAP-negative tumors according to the Kaplan–Meier survival curves. Moreover, FilGAP expression was shown as an independent prognostic factor for FL, but not DLBCL, by Cox regression analysis, suggesting that FilGAP-positive FL may constitute the unique subtype with aggressive clinical course. It is likely that high FilGAP expression causes establishment and maintenance of amoeboid features in lymphoma cells, which induce adaptation of their shapes to squeeze through pores in the extracellular matrix, resulting in the accelerated progression and the extensive dissemination. Further studies to clarify these points are clearly warranted.

In conclusion, the present study clearly provided evidence that FilGAP is frequently expressed in B-lymphocytes. Moreover, FilGAP appears to be useful for predicting the behavior of B-cell lymphomas, in particular FL.

## Supporting Information

**Figure S1.** Perinuclear Rac1 grades and scores in (A) lymphocytes and (B) lymphomas. Upper: Relationship of Rac1 immunoreactivity between the perinuclear grades and the cytoplasmic (Cyto) scores. Lower: Perinuclear Rac1 scores in normal lymph nodes (left) and lymphomas (right). The data shown are mean ± SD.

**Figure S2.** Progression-free survival (PFS) and FilGAP expression. The 5-year PFS of stage I–IV FL (left) and GCB-type DLBCL patients (right) based on FilGAP expression.

**Figure S3.** OS of stage I–IV FL patients based on integrin *β*2 (left), ECT2 (middle), and cytoplasmic Rac1 expression (right).

**Table S1.** Summary of malignant lymphoma cases investigated.

**Table S2.** Relationship between Rac1 IHC and perinuclear scores in normal lymphocytes and malignant lymphomas.

**Table S3.** FilGAP expression and clinicopathological factors in malignant lymphoma.
