# Supplementary figures and images for: FilGAP, a Rac-specific Rho GTPase-activating protein, is a novel prognostic factor for follicular lymphoma
Source: Cancer Med. 2015 Jan 29;4(6):808–18. doi: 10.1002/cam4.423 (PMC4472203; doi:10.1002/cam4.423)

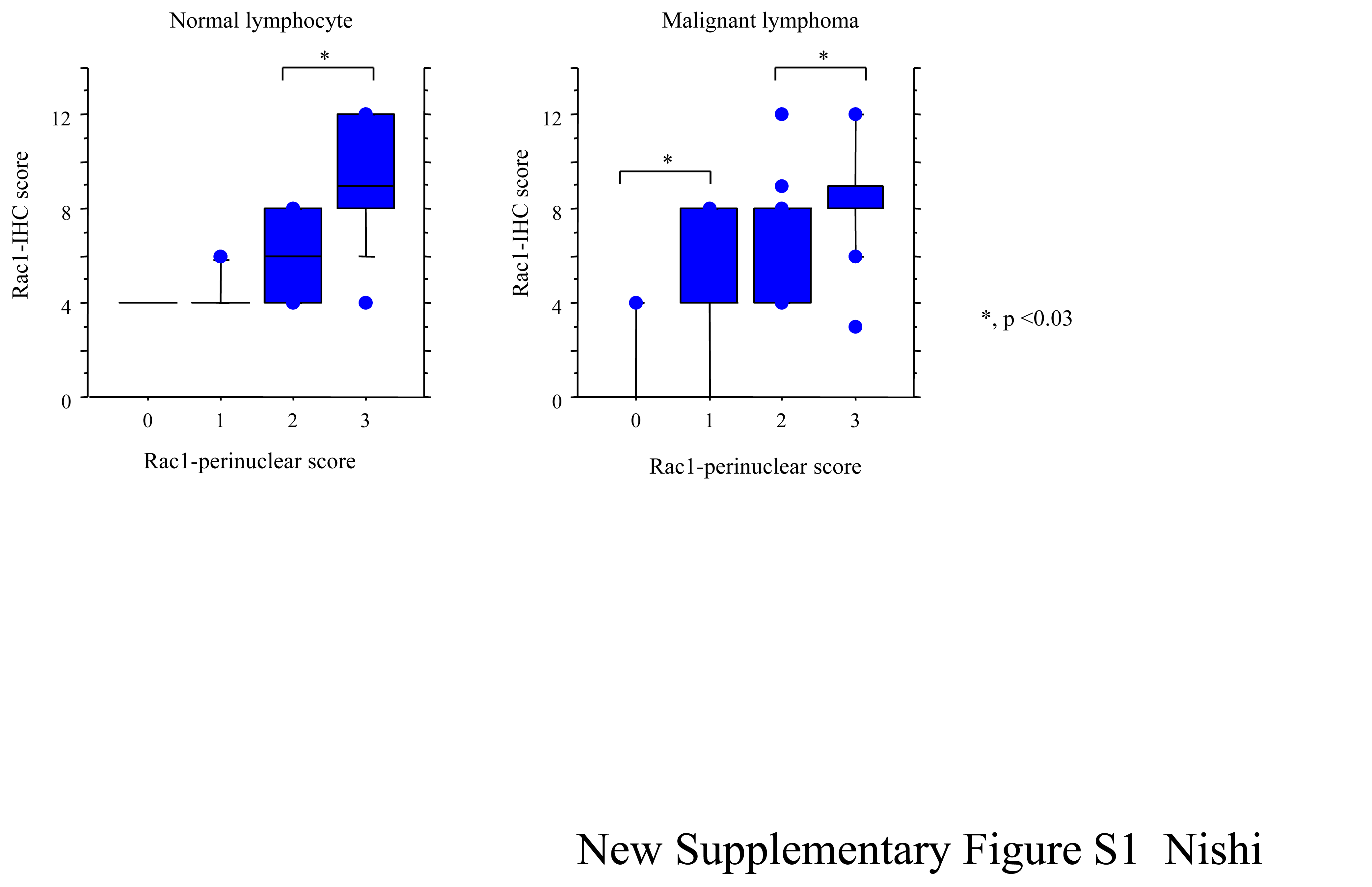

Supplement: Supplementary file 1 [file cam40004-0808-sd1.tif]

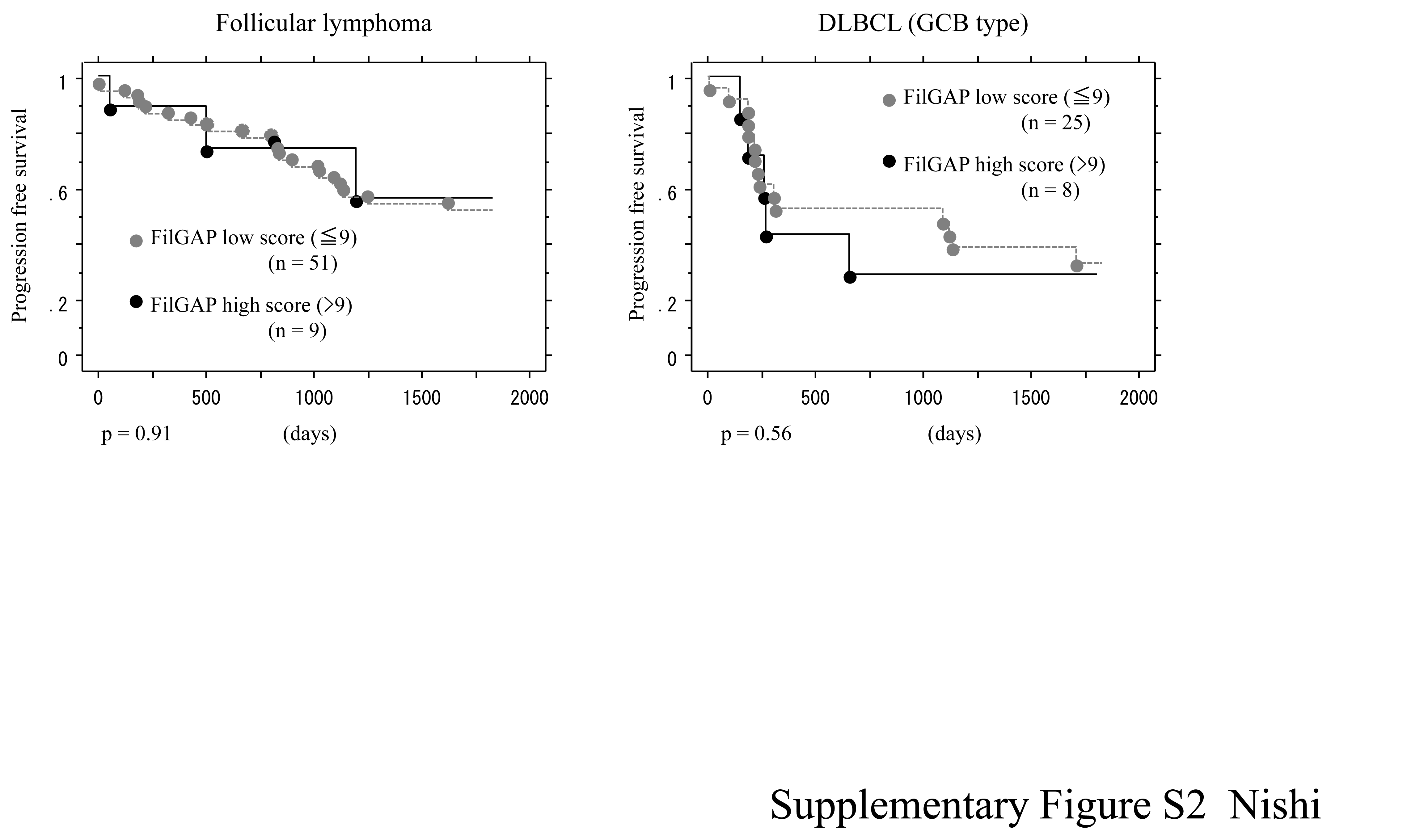

Supplement: Supplementary file 2 [file cam40004-0808-sd2.tif]

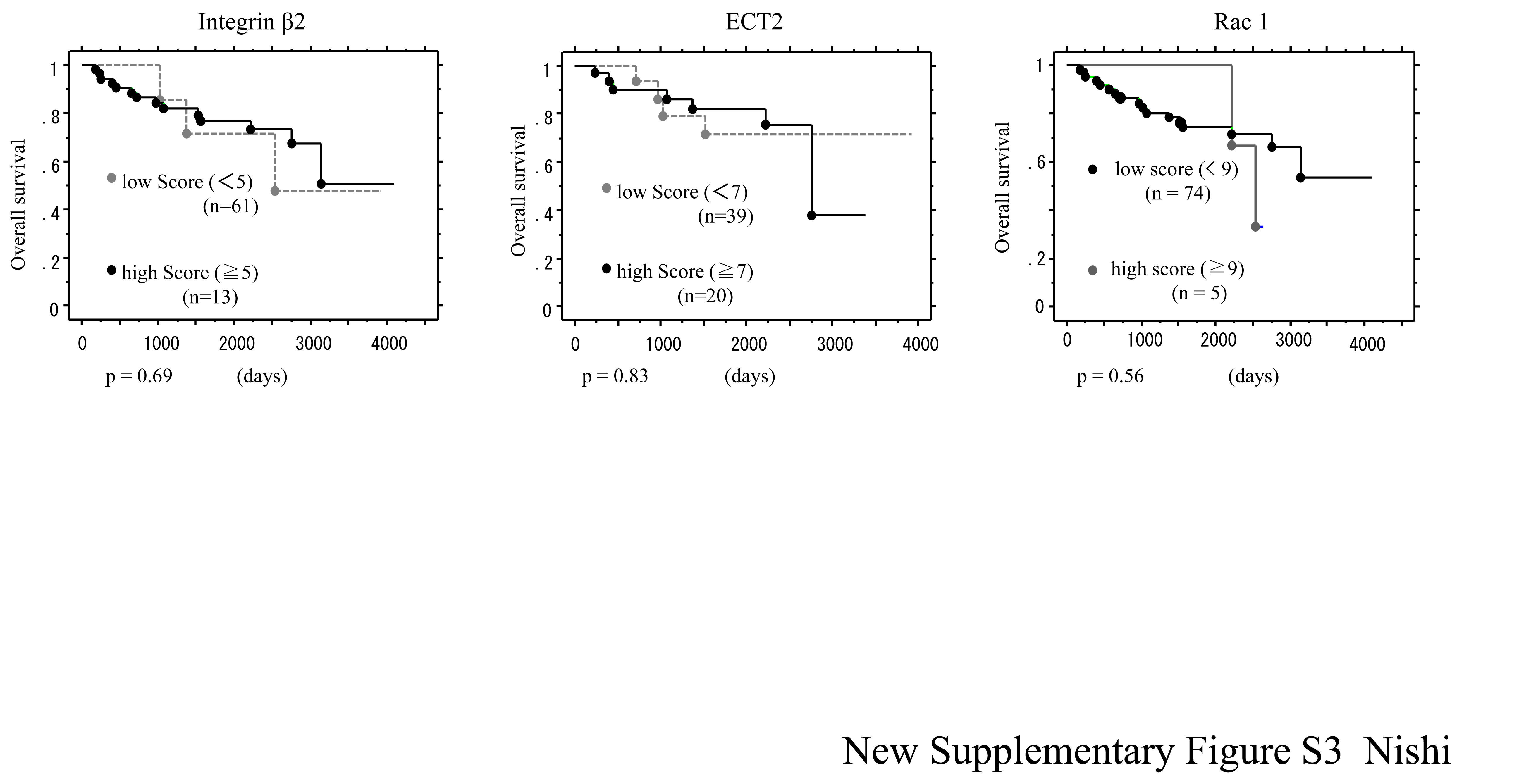

Supplement: Supplementary file 3 [file cam40004-0808-sd3.tif]
